# Medico-Legal Trial

**Published:** 1859-07-01

**Authors:** 


### MEDICO-LEGAL TRIAL.

### Disputed Will.—Plea of Unsound Mind.

COURT OF PROBATE, Westminster, May 5th.

(Before Sir C. Cresswell, the Judge Ordinary, and a Special Jury.)

Terrington *v.* Terrington and Johnson.

This was a suit to try the validity of the will and the testamentary capacity of the testator, one William Terrington, of Emneth, near Wisbech. The plaintiff, Zachariah Terrington, the executor, averred that the testator at the time of the execution of the will was of perfect sound mind, memory, and understanding. The defendants, who contested the will, were Mrs. Mary Terrington, the mother of the testator, and Mrs. Hannah Johnson, his sister, and they averred by their pleadings that the testator was not of sound mind, memory, and understanding, and that the execution of the will was obtained by undue influence exercised over him by Zachariah Terrington.

Mr. Serjeant Pigott and Dr. Tristram were counsel for the plaintiff; Dr. Phillimore and Mr. D. Keane were for the defendants.

It was stated by Mr. Serjeant Pigott, in his opening of the case, that the plaintiff, Mr. Zachariah Terrington, was uncle of the testator. The will was made on the 19th of January, 1859, by Mr. Ollard, Solicitor, of Upwell, near Wisbech, and the testator died on the 24th of that month. By the will he bequeathed a legacy of 19*l.* 19*s.* to his uncle; he bequeathed the interest of a sum of 200*l.* to his sister, a Mrs. Desborough, in America, and at her death the principal was to be divided amongst her children; and the residue of his property, which consisted of about thirty acres of land, and some 300*l.* or 400*l.*, he bequeathed to the children of his uncle, Zachariah Terrington. The learned counsel detailed the history of the testator's life, to show how it was he had been induced to exclude his mother and others of his family from any benefit under his will. It appeared that at the early age of two or three the testator was sent to live with his grandfather, where he remained until he was fifteen, the grandfather having in the meantime attended to his education, and in fact brought him up as one of his own children. When he had attained the age stated, he was sent home with the view of his providing for his own livelihood. It was alleged, however, that the boy was treated with the utmost neglect by his mother, and after remaining under the parental roof about a week, he set off roaming about endeavouring to earn a living by his own labour. Sometimes he obtained a job at one relative's and then at another's, but was eventually so reduced that he had to apply to the guardians for relief. Subsequently he worked a passage out to America and back, and after that it seems he resolved to try his fortune in America, and accordingly in the year 1850 he went out and settled in that country. In 1852 his brother John died and left him the property now in dispute. The testator continued in America until 1857, when he returned to England, and first made his way to his uncle Zachariah's house, and remained there some short time. He was persuaded to go and live with his mother, but it was stated that he experienced the same want of maternal affection on her part that drove him away from home at the former period of his life, and in addition to this he had got the impression that she wished to poison him. He accordingly returned to live with his uncle. This was on Monday, the 17th of January, in the present year. On Wednesday, the 19th of the same month, he went to Upwell and made his will, and on the following Monday he died. All the circumstances will be found fully set forth in the evidence.

Mr. Ollard, attorney, Upwell, Norfolk, examined by Serjeant Pigott: I had no acquaintance with the testator before the 19th January, 1859, further than having seen him occasionally. I have known his uncle Zachariah for many years. On the 19th of January the testator came to my office accompanied by his uncle. It was between ten and eleven in the morning. William Terrington said, "I wish to make my will." I said, "Would you not like to be alone?" Upon my saying so, Zachariah Terrington got off his chair, and the testator said, "No, I don't wish you to go; I wish you to stay; I mean to appoint you one of my executors, and I would rather you stayed and heard." The testator appeared to me to be faint, and I said to him, "Would you not like to have a little ale?" He replied, "Walking is an exertion to me, and I am tired; but I am determined not to take a drop until I have made my will." I then said to him, "What property have you?" He said, "I have about thirty acres of land, and about 600*l.* which I have lent to my brother-in-law, Johnson, on a note, and a few other things of small value." I then said, "How do you wish to leave it?" He said, "I wish to leave the interest of 200*l.* to my sister in America for life, and then to her children; 20*l.* to my uncle Zachariah; and the remainder of my property to be divided amongst his children." I said, "Who do you wish to be executors?" He said, "I wish my uncle Zachariah to be one, and I should like you to be the other." I said, "I hardly like to be so. What makes you choose me; you don't know me?" He said, "I don't know you myself, but I have heard many speak of you, and I have a particular wish that you should be my executor." He said, "I don't want to trouble you in the matter, but I want some one that I can rely on to send the money to my sister in America regularly." I said, "If that is your object I don't mind." I then said, "Trustees are not entitled to charge; and as you are no interest to me I should object to be a trustee and not charge." He said, "Oh, I don't want that; I wish you to be paid all your usual charges." I said, "Very well; I'll put a clause in the will;" and he said, "That's right," or "All right." I then commenced writing the will. After I had written a line or two, I said to him, "You seem to me in a very feeble state of health." He said, "I am not well; I am subject to bleeding, which weakens me a great deal." I then said, "I am quite satisfied with your perfect comprehension; but seeing your state of health it would relieve my mind that you should see a medical man." He said, "I have no objection. I wish everything done to make my will secure; send for anybody you like." I called my clerk, and directed him to bring the first medical man he found at home. Whilst the clerk was gone I said, "Are you quite determined to leave nothing to your mother and your other sister?" He said, "Not one farthing." I said, "It will excite a very bitter feeling in your mother's mind." He said, "I have no doubt of it; but I expect I have a right to do what I like with my own." Upon that I went on writing the will until Mr. Tubbs came. When Mr. Tubbs arrived I said to him, "This is Mr. William Terrington, sir; he has instructed me to make a will, and he has given me instructions with very great clearness, but he seems to me in a feeble state of health, and there will probably be dissatisfaction caused if he should die; and I therefore should be glad if you would examine him yourself and say what you think." Mr. Tubbs sat down by him and asked him his age, or something to that effect, and then said, "Are you in the habit of drinking?" The testator said, "I am sorry to say I am; but I have not taken one drop this morning, for I was determined to come solid and sober to make my will." Mr. Tubbs then went on speaking to him, and I went on writing the will in hopes of being able to finish it in time for Mr. Tubbs to sign it. Before I had finished writing, Mr. Tubbs said to me, "He is as fit to make a will as I am." I said, "So I thought myself, but still it is a satisfaction to have your opinion. Will you stay and witness it?" He said, "I am in a hurry, and cannot; but you can go on with perfect propriety." He then left, and I went on and finished the will. After I had finished it, I read it over to the testator. He put several questions upon it as I proceeded. He asked whether his lands would go under the general description, and other questions all relevant to the matter; and when I finished he said, "You have got it quite right." I then went for Mr. Stuck, my nearest neighbour. Mr. Stuck came, and I called Balding, my clerk, and I said in their presence, "Mr. Terrington, you have heard this will read, does it correctly express your desires?" He said, "Yes." I then said, "Will you then execute it, and desire these two gentlemen to attest it?" He said, "Yes." And he then signed it, and Mr. Stuck and Mr. Balding signed it in his presence. When that was done he said, "Uncle, I wish you would kindly fetch the gig from the Five Bells, it will save me the trouble of walking." His uncle went, and he stayed with me and said, "You offered me a glass of ale, but I would not take it before I signed my will; now, I should be much obliged if you would let me have it." I rung for a glass of ale, and he leant back in his chair and said, "Now I feel very much relieved now that I have made my will; and I should have been very much annoyed if my mother or Mrs. Johnson got a farthing of my money." I said, "What is the reason you seem to dislike your mother so much?" He said, "Oh, she has never been a mother to me." I said, "You have been living with her for some time, have you not, though?" He said, "Yes, but I have never been comfortable there; but I have long been wishing and intended to leave. I rather hastened away, and have left her now for good." I said, "What hastened you away?" He said, "Why, the servant girl told me something which I did not like." I said, "What was it?" "She said she saw my mother put something in my drink when I was not there. She told me she threw it away and put me some fresh drink." I said, "Oh, your mother could not have intended to have hurt you." He said, "I don't know that she did; but you do not know my mother as well as I do." He said he was going to live at his uncle's a little while, and then he intended to take a cottage.

Did he appear to you to understand what he was talking about?—Most perfectly so.

Was his conversation all rational?—Perfectly so in every respect.

Did he say anything to indicate an unsound state of mind in any respect?—Not one word.

Cross-examined by Dr. Phillimore: I would not undertake to say the conversation I have detailed is verbatim, but it is the substance of what he said. I know Mr. Bays, conveyancer, of Wisbech. He came down to have the will read on the part of Mr. Johnson, at the funeral. He did not say that he was surprised I made a will for such a person as that. Mr. Bays told me the will would most likely be disputed, and I said, if that is so, I shall not act as executor; and I did not much like being made executor at all, but the testator pressed me. I did not know the testator before he went to America. Zachariah, the uncle, I have known many years; he keeps a public-house at Emneth.

Do you not know that it is a disreputable house?—Certainly not; on the contrary, I think it is a most respectable house. I was never there with Mr. Tubbs. About a fortnight ago I met the witnesses there and took their evidence down in a room. During the whole of the conversation with the testator the uncle never said a word. This was the first intimation that I was required to make the will for him. The uncle had not said a word to me upon the subject. I know Mr. Metcalfe is solicitor for a portion of the family, but there is not what I call a family solicitor. I know Mr. Wilkin. I do not know that he refused to make the will. The testator answered all my questions very clearly, and gave his instructions as clearly as either you or I. I do not know that I should have suggested sending for a medical man had I not known Mrs.

Terrington's peculiar character. I thought she would be sure to dispute the will if there was the slightest occasion for it. I was not at all aware of the habits of the deceased beyond what he himself told me.

Mr. James Francis Balding examined by Dr. Tristram: He said he was clerk to Mr. Ollard, and recollected the testator coming to the office on the 19th of January last. Witness was present the whole time Mr. Tubbs was in the room, and he was one of the attesting witnesses to the will. The testator signed in the presence of witness and Mr. Stuck; and the attesting witnesses signed in each other's presence and in the testator's presence. Witness heard Mr. Tubbs say, "He is as fit to make a will as I am." The testator appeared to be as fit to make a will as any one he had ever seen in the office.

Cross-examined by Dr. Phillimore: He judged of the testator's competency to make a will from the straightforward manner in which he answered the questions that were put to him.

Mr. James Reynolds Stuck examined by Dr. Tristram: He lived at Upwell, and carried on the business of a grocer and draper. He never saw the testator until he attested the will at Mr. Ollard's office. The testator appeared calm and collected, but very unwell. He saw nothing to indicate that the testator was not capable of making a will.

Cross-examined by Dr. Phillimore: Witness knew nothing about the family. He was in the room from ten to twenty minutes. He put no questions to deceased himself.

Zachariah Terrington examined by Serjeant Pigott: I am the uncle of the testator. I live at Emneth, near Wisbech. I am a publican and a farmer. The house is called the Hungate Inn. I farm about 120 or 130 acres of land. I possess land of my own besides. My father's name was John Terrington. The testator lived at my father's house some time. He was between two and three years old when he first came there. He continued at my father's till he was about fifteen. He was thirty-two when he died. I lived at home while he was with us. My father sent him to school, and when he was fifteen he was sent home to his father and mother. He lived at home a day or two, until he fell out with them, and then he went roaming about from one place to another. When roaming about he was sometimes along with me and sometimes at Mr. Howlett's, working for us. I had a farm of my own then. He would stay a fortnight or three weeks with me at a time. Mr. Howlett married his mother's sister, and was a publican and a farmer in the Fens. The testator was not able to get a livelihood; and on one occasion, in 1849, he applied to the parish. I was not present when he applied to the guardians, but I heard my brother say one of the guardians had given him an order. The testator went to America, working his passage out, and then he came home again. He returned to America, and was away six years. Before he went to America he borrowed money of me several times, as he was in poor circumstances. His father died about 1852, and left the testator a sum of 20*l.*, which I believe my father sent out to him. I produce letters from the testator, written while he was in America. [One of the letters, dated April 25th, 1852, was read, acknowledging the draft and giving the testator's impressions of American character. He described their extreme inquisitiveness and their proneness to relieve strangers of their money, "either by hook or by crook;" and he added, "You must not get drunk here and lie on the seats as I used to do in those old publics on the smees or fens." Other letters were read.] I remember his brother John dying in 1857. It was soon after that the testator was sent for to return home. I never wrote to him while he was in America. My father has been dead about three years. The testator arrived home from America in November, 1857. He came to my house the day after he landed, and stayed a week with me. He was taken ill the day he arrived, and Mr. Wallis, a doctor, was called in to attend him. Mr. Wallis is since dead. The testator was in bed all day. I

believe his illness came through drink. He went to bed as soon as he came. He got up the next morning. The doctor visited him twice, but only saw him once. He had some medicine the first time the doctor came. While he was staying with me that week he amused himself by walking about with me on my business. He talked about farming and about America, and appeared to be well. He went to see his mother on the Saturday in consequence of my expressing a wish to that effect. He went off to see her by himself, but I met him on the road and accompanied him. We met Mrs. Johnson on the road in a gig, going to market. I was walking by the gig, and we saw the testator coming. She said, "That's my brother." I said, "Yes, I think it is;" and she said, "I'll drive on." She drove away, and after she had passed him he came up and said, in reference to her conduct, "She's a nice sort of a sister; she didn't speak to me." He asked me to go with him to his mother's, and I turned back and accompanied him. While we were going along the road, he said my wife wanted him to go and see his mother. He stayed at his mother's about ten minutes. I do not know whether it was a friendly meeting, as I stopped outside. I remember his asking me to go to Mr. Metcalfe's about his brother's affairs. He said, "Will you go and see me righted about my money?" I said "Yes." He told me his money was all at Mr. Metcalfe's office. Mr. Metcalfe said it was not the proper time to receive it. At the end of the week he went to live at his mother's, and remained there till January, 1859. He visited me two and three times a week, and on one occasion stayed all night. I remember his coming on Monday, the 17th. He came with Mr. Forth, the baker, in his cart, and he said he and his mother had been falling out, and he would not stay any longer with her. He asked whether he might keep along with me. I said he might as long as he liked, and when he got tired he might leave. He then asked me if I would go along with him and make a will the next morning. I said I would if I was not busy. I was busy the next day and did not go with him; but I went with him on the following day, Wednesday. There was a man named Diggle in the house when he asked me, and the testator wished him to go along with us. I drove him to Upwell to Mr. Ollard's office at his request. He had seen Mr. Ollard's name in the newspaper. He said, "We'll go to that little man at 'Well." I said, "You mean Mr. Ollard?" and he said "Yes." He said he would not go to Mr. Metcalfe because he could not get his money from him. We conversed but little on the road. He borrowed some money of me as we went along, for the purpose of paying Mr. Ollard for making the will. I remained in the room while the will was made at his own request. I remember my nephew speaking to me about having bought a hoe to amuse himself with it, and telling me that his mother complained of his eating too much, and wished him not to use the hoe. He also said his mother had a cask of liquor in the house, and wanted to sell it out to him by the bottle. He drank a good deal occasionally. I have never seen him intoxicated. I remember his mother coming to my house when he returned from America, but I was not at home at the time. He was taken ill on Sunday afternoon. I asked him if I should fetch a doctor. He said no; if he was worse he would let him know. I saw him again that day. He said he neither felt ache nor pain. I again spoke to him about the doctor, and he said if he felt worse he would let him know. The next morning at nine o'clock, after I came in from the farm, my wife told me he was a little worse. I went up and finding he was much worse, I put my horse to and went for Mr. Tubbs. Mr. Tubbs came between eleven and twelve, but by that time he was dead.

Cross-examined by Dr. Phillimore.—I did not send for Mrs. Bradley on Saturday to nurse him. Mrs. Bradley is a charwoman. She came on Saturday and sat up with him all night, for the purpose of giving him a little drink when he wanted it—wine-and-water and gin-and-water.

The Judge.—Why did Mrs. Bradley sit up with him on Saturday night to give him drink?—Because there was no bell-pull.

But how was it that she sat up with him that night and not on Friday night?—Because "my missus" could not do so.

But was it necessary that anybody should sit up with him at all?—I don't know any reason particular. He might be a little worse—he was not much worse. He was not bad the day before.

How came you to swear that you did not know anything of his illness until Saturday afternoon?—He appeared much the same as usual.

Cross-examination continued.—My son always slept with him except one night, and then my son refused to sleep with him because he wetted the bed. He did not tell me he was frightened to sleep with him because he raved so. When he was in America I am not aware that his father and mother sent him money.

Was he not an habitual drunkard before he went to America?—He used to drink a little; he used to get drunk sometimes, and sometimes he did not.

Was he not generally drunk?—Yes; he used to get drunk a goodish bit. He returned in 1857. He was brought to my house in a cart. He was not capable of taking care of himself. I believe he was a little bit drunk. During the week he was at my house he was never drunk but once.

Upon your oath, was he not covered with lice, owing to his drunken and careless habits, when you took him to his mother's?

No. I did not say to his mother, "Unless he is well looked after, he will be lost;" nor did I tell her that he had been found in the road with his clothes down and his shirt over his head, and his body covered with dirt. I believe he was so found on the road, and he was brought in that condition to my house. His mother kept an inn called the Jolly Farmers at that time. I do not know that in April, 1858, the testator was at a public house called the Chequers, at Emneth. That house is kept by my niece. I never went to Mrs. Terrington, the mother, to tell her of what her son was doing at the Chequers, or what was being done to him. I never said they were going to get him to marry that girl (meaning Thrasher's sister), and that they were going to take him to Lynn to get him to turn his money over to her, and that when they did they would soon turn him off, and fly about spending the money. I know Mrs. Terrington's (the mother's) servant. I did not know her name was Mary Fundry. I never said to her, "I am sure he is not in his right mind; he never was in his right mind all the time I knew him." Nor have I said that I had to get up four or five times in a night to bottle off brandy for him, and that I had to lock him up. I never said to Mary Fundry that I was sure his mother had poisoned him. I swear distinctly that I never said to her that the deceased had told me that his mother had poisoned him. He had fits when he first came home, but not lately. The fits lasted about two or three minutes. He would lay quite stiff, with his hands contracted and his mouth wide open. I gave no notice to Mr. Ollard beforehand that we were coming to make the will. My nephew began the conversation by asking me if I would go with him. He never said how he was going to leave it. I never said anything to him about leaving it to his mother and his sister, or to his sister in America. He never said anything about having asked either Mr. Metcalfe or Mr. Wilkin to make his will. I heard the solicitor ask him if he was ill. I did not say anything. I knew he was ill from bleeding; I did not know that he was ill from excessive drinking. I do not know that excessive drinking causes death. I never said to Mary Fundry anything about the difficulty of getting his clothes off the night he made the will. His legs were not so contracted that he could not get his clothes off. Mr. Tubbs gave the certificate of the cause of his death. Mr. Tubbs saw him at Mr. Ollard's, and he did not see him again until after his death. I did not tell Mr. Tubbs the cause of his death. After he came back on the Monday from his mother's he was not once drunk up to the time of his death. He was not drunk on the Tuesday; he had about a pint of beer that day. He had no spirits. He might have had two pints of beer. On the Monday he did not have above one pint at my house. Nobody sat up with him on Monday and Tuesday nights. My son slept with him. I was not present when my wife told Mr. Johnson my boy dare not sleep with him on account of his raving. I do not know that on one occasion he was so drunk that he fell down in the pigsty and the pigs walked over his body. He could not hold his water; he stood trembling, and the water fell from him. He was sometimes covered with his excrement, which fell from him. I did not tell Mr. Ollard any of these things. I did not think that Mr. Ollard ought to have been made acquainted with his condition. He took no liquor on the Wednesday morning when he went to get his will made. The reason why he took none was because he was ailing from bleeding at the nose and mouth in bed. He came down with his mouth all covered with blood.

Re-examined by Mr. Serjeant Pigott.—The woman Bradley occasionally did work at my house. I never asked her to sit up with him, and I am not aware that my wife asked her to do so. Mrs. Terrington's servant (I did not know her name was Mary Fundry) came to hear the will read at the funeral. He had no fits in my house during the last week before his death.

By the Judge.—When he came back from his mother's house the last week before his death, he said the girl had looked through the keyhole and seen her mixing something in a glass.

Mr. William Tubbs, examined by Serjeant Pigott.—I have been in practice in Upwell upwards of twenty years. I remember being sent for on the 19th of January to go to Mr. Ollard's office. I there saw the testator. Mr. Ollard told me why I was sent for. He said: "I have sent for you to see this Mr. Terrington, although I am perfectly satisfied as to his capability to make a will." After passing Mr. Ollard, I questioned the testator as to his past life. He had the appearance of taking a little drink, which he acknowledged. I said, "Are you rather fond of taking a little drink?" He said, "Yes." I gave him a multiplication-table to test his capability. I asked him how much twice seven was, and three times seven. All I can say is, that he answered all my questions in a straightforward manner. He said he had come to give Mr. Ollard instructions to make a will, that he came sound and solid on purpose, and had taken no drink that day. I felt his pulse and looked at his tongue. His pulse was slow and jerking—what we call a hæmorrhagic pulse. I sat down and conversed with him for about a quarter of an hour. I am satisfied from what I saw of him that he was then endowed with full consciousness and perfect reason, and quite competent to dispose of his property. I detected nothing to indicate an unsound state of mind. I examined his mouth, and found symptoms of a scorbutic humour in the mouth, as well as of raising blood. The inability to retain water may arise from other causes than from excessive drinking. Epileptic fits and delirium tremens do not necessarily produce mental incapacity. I said I was perfectly satisfied as to his competence to make a will, and I left. I was sent for about a week afterwards to his uncle Zachariah's, and found him dead. I gave a special certificate, which I derived from the examination I made on the day I saw him at Mr. Ollard's office. His condition then was consistent with the nature of his death. I apprehended that he might be suddenly carried off from syncope, the languid circulation being insufficient to drive the blood to the head.

Cross-examined by Dr. Phillimore.—I gave the certificate to Mr. Terrington, stating that if that certificate was not satisfactory it would be necessary to have an inquest. I conceive syncope to have been the cause of his death. I found that opinion upon the languid circulation he had, and from the circumstance stated by his attendants. They stated they were going to give him some drink, when he turned up his eyes and died. I have no doubt that the deceased was of full consciousness and reason when I saw him, and I founded my opinion as to that upon the general conversation with him. I did not ask him about his property or about his relatives. I recollect asking him what day it was, and he told me correctly. I did not ask him about his former illnesses. I was not aware he had had epileptic fits; had I known it, it would not have altered my opinion. Epilepsy proceeds from a variety of causes, not entirely from drink. Had I been aware that the deceased was subject to epileptic fits, and was an habitual drunkard, it might have modified my opinion as to his full consciousness and perfect reason. I have performed an operation under the influence of mesmerism.

You are a believer in mesmerism?—Of course I am. I could mesmerize you in a very short time. (Laughter.) I beg to say that I have cured cases of insanity by mesmerism.

The Judge Ordinary said there was no rule of law, whatever differences of opinion there might be upon the subject, that because a person believed in mesmerism he was insane.

Mr. Serjeant Pigott.—Nor that a witness is not to be believed.

Cross-examination continued.—I never said the testator told me that his mother had poisoned him. What I said was, that I heard a man, who was getting his dinner at the house when I called there on the day after his death, say the deceased had said that if he had remained with his mother he would have been muddled out of his life. By his appearance at Mr. Ollard's I thought he was fond of drinking. His flesh was pale and emaciated.

By the Judge Ordinary.—Drinking sometimes produces a rubicund colour and sometimes a pale colour; the pale colour denotes a more advanced stage of intemperance.

Cross-examination continued.—I should say he had the appearance of being a confirmed drunkard. His observation that he came sound and solid struck my attention. He had symptoms of pectoriloquy, or talking in the chest. The inability to hold water is a sign of an affection of the brain, but it might also result from a calculus in the bladder.

Re-examined.—With inability to retain water there may be perfect soundness of mind.

Mrs. Mary Terrington, examined by Dr. Tristram.—I am the wife of the plaintiff, Zachariah Terrington. I have known the testator for the last twenty-seven years. I recollect him going to school in 1840. After he left school he went home, and after he left home he was with me the whole of one summer—that was some time before he went to America. On his return from America he came to our house, and was brought in a cart. He was very dirty indeed, and my daughter washed him, and put him one of my boy's shirts on, and put him to bed. We then sent for Mr. Wallis. Friday he was ill. Saturday he got up as usual, and I advised him to go to his mother. A day or two before the 17th of January last I received a message from the testator. I sent word back that he might come and stay with me as long as he liked. He came on the 17th, about six in the evening. He was quite sober. He had something the matter with his legs then, so that he was obliged to walk on his toes. I heard him ask my husband and Mr. Diggle to go with him to make his will. He was sober on the 18th, and was down in our little grass-field. On the 19th he bled very much in the morning. He got up about nine. I washed his hands and face, and combed his hair out, for he was smothered with blood. He told me he was very often so. On Thursday, the 20th, he was up all day. On the Friday and Saturday the same. On the Saturday night I thought he was worse than he had been. I said I would not let my little boy sleep with him, and I sent for Mrs. Bradley to sit up with him. I never heard him complain during Saturday night. On Sunday I proposed to send for a doctor. He said, "I

won't have him fetched; I shall know when I am any worse, and I will call you." On the Sunday he had some wine-and-water. On the Monday morning he was worse. I gave him wine-and-water three or four times in the course of the morning. When Terrington came in from the fields I told him the testator was worse, and told him to go for Tubbs. He got his horse, and, without waiting to put on a saddle, he went for Tubbs.

Cross-examined by Dr. Phillimore.—I remember on one occasion calling at Mrs. Terrington's after she left the Jolly Farmers, and having some conversation with her about the deceased. I do not recollect saying that he was not in his right mind. I could almost swear I did not. He was constantly in a state of the most horrible filth when he was with us. I have complained of his wetting the bed. He was subject to that when he was a child. I have often heard him say that his mother tried to poison him, but I always hushed him, and told him it was all nonsense. I have heard him say so several times. I never told Johnson that my boy could not sleep with him any longer on account of his raving. I never said we would not let him have any drink because Mr. Tubbs told us not.

Re-examined by Mr. Serjeant Pigott.—I never saw Mr. Tubbs. He said the girl told him his mother had tried to poison him. His mother saw him at our house after he arrived from America.

By the Judge Ordinary.—I sent for her the same day. She came and saw him. He was then in bed. I heard him tell her she had never been a mother to him. She said, "How can you say so." He made answer, "You never was a mother to me; my grandfather's old housekeeper was my mother." If it had not been for me and Mrs. Henry Terrington he would have got out of bed and knocked her down. I held him in bed, and Mrs. Henry Terrington took the mother into my room. I first recommended him to go to his mother. I recommended him on the Friday to go and see his mother. He was sensible, and able to help himself. My daughter washed him because he was dirty. I sent for the mother because I thought she would like to see him. He was tolerably sober when he wished to use violence towards his mother. He always had a strong feeling against her. The next morning I persuaded him to go, when, for what I know, he might be in a little milder temper.

The Judge Ordinary.—We can all judge about that.

Thomas Bellinson, examined by Mr. Serjeant Pigott.—He lived at Walsoken, and was a farmer and innkeeper. He knew the deceased before he went to America, when he was greatly in want. He got into debt at his house to the amount of twenty-eight shillings, and as soon as he returned from America the last time he called and paid the money. He understood what he was about just as well as witness. He talked about America, and about old times. He had a long memory, and told witness things he could not recollect himself. Witness saw him after that about once a week. He took a glass occasionally at witness's house. Witness had seen him walking about with the superintendent of the fens and other persons, and had frequently conversed with him. There was nothing to indicate in the slightest degree an unsound mind. Witness saw him about a fortnight before his death, when he came to his yard and said his mother had been trying to poison him. Witness replied, "You should not say so; you'll get your mother into difficulties." He asked witness what he had better do, and the reply made was, "I would neither eat nor drink in the house again if I thought so." He said the servant girl told him. He had been tipsy that day, but he had laid down to sleep, and had got over it. When he was talking to witness he appeared "quite undisturbed" in his mind. He walked with a stick.

Cross-examined by Dr. Phillimore.—The money testator owed him was for beer. Witness saw Mr. Ollard at Zachariah Terrington's beer house, and told him he did not wish to be a witness. He never told Johnson that he thought the deceased was crazy: he never said such a thing. Witness could not answer the question, whether he believed at the time that the mother had been trying to poison him—he should think the mother knew better than that. By "undisturbed in his mind," witness meant "disturbed." He had seen the deceased drunk, and so he had a great many men, and no fools either. He might have had some conversation with the testator about his property.

Re-examined by Mr. Serjeant Pigott.—About a month after his death his mother sent for witness and said she was a poor widow in difficulties, and she wanted him to do her a kindness. She said, "I want you to go and say that you think my son was not in his right mind," Witness replied, "I cannot say any such thing. I believe he was as right in his mind as I am." I said, "Johnson was a drinking man; Howlett was a drinking man; and they made the best of bargains and made lots of money; therefore, if a man is capable of getting money being drunk, he is capable of giving away money being drunk." I added, "The last word I heard your son say was that you had been trying to poison him." She said, "Nonsense, I used to give him a small pill of opium at times, when he had a craving for it." She said she would show him the size of the pill, and she then showed him a pill in a tin-box.

Cross-examined by Dr. Phillimore.—Before he went to America he was accustomed to smoke and chew tobacco.

William Wightman, examined by Dr. Tristram.—He was a farmer and innkeeper. He was acquainted with the testator, and had known him for fourteen years, and had played and worked with him. On his return from America, he paid witness a debt of 15s. or 16s., which he contracted before he went to America. Since his return witness saw him repeatedly. A few days before his death, he said his mother had tried to poison him. The servant girl told him so, and that his mother had mixed up some several times, and he attributed his bad legs to what she had given him. The testator talked rationally upon subjects that had occurred many years before.

Cross-examined by Dr. Phillimore.—Part of the debt was for drink and part for victuals.

Charles Diggles, examined by Serjeant Pigott.—He was a farmer at Emneth, and knew the Terringtons. Before the testator went to America he lent him 7s. He was fond of drink. Since his return from America he had seen him regularly once and twice a week. He always talked as well as another man could. On the 17th of January witness saw him at the Jolly Farmers, when he said, "Have you heard anything about this pill concern?" Witness said he had heard a little about it, but he never took any notice of it. He rejoined, "There's a large pill here which mother was going to give me." Witness asked, "What was she going to give you that for?" "Well, I don't know," he said, "she sent the gal with it." He added, "That arn't all, Charles; the gal was looking through the keyhole, and she see my mother mix something up in a glass, and she gave it to the gal and told her to bring it to me, and the gal told me she flung it away." He then said, "You shall have that little money as soon as I can get hold of it, but I shall have a row with my mother and Mr. Johnson, for they will not let me have enough to pay you with." Forth then came in, soon afterwards, and the testator went away with him in his cart. He had heard there was a pill in the house, but he did not see it. On the same occasion the testator complained of his infirmities, and gave that as a reason why he stopped so long at his mother's. In the evening witness went to Zachariah Terrington's, and the testator asked him to come and sit by the fire. He then said he wanted witness to go with him and his uncle Zachariah to 'Well, as he wanted to make a will. Witness said "he would rather have nothing to do with it, as he was no scholar." About two nights afterwards witness again saw him, when he said he did not feel very well, and very soon afterwards he called to his aunt to assist him upstairs. The calves of his legs were affected, so that he was obliged to walk on his toes. He always spoke very badly of his mother, saying that before he went to America she would not let him go nigh the house. Witness had seen the tears fall from his eyes when he had been speaking about it. He always talked rationally and as well as anybody else.

Cross-examined by Dr. Phillimore.—The testator said the pill was kept at the Jolly Farmers, but witness had never seen it. What he said about the pill in the morning did not influence him in the evening in refusing to go and attest the will.

William Forth, examined by Dr. Tristram.—He was a baker and grocer at Emneth. He knew the deceased for twenty years. He had frequently seen him since his return from America. He took him to Zachariah Terrington's on the 17th of January. Witness had heard the report about the mother trying to poison him at the Hungate, and when he saw him at the Jolly Farmers, the testator declared it was true. He never said anything in favour of his mother. On one occasion he said he would make a will as soon as ever he left the old b———, and that when he did make his will he should think of his uncle Zachariah, of whom he always spoke very highly, saying he was the best uncle he had. Since his death witness had talked with the mother about the will, and she said she did not think it was right to leave the money out of the family; and she said if he was called as a witness at the trial he need say nothing on either side.

Cross-examined by Dr. Phillimore.—The first he heard of the report about the poisoning was from Mrs. Zachariah Terrington, who, at the same time, said that he might go and live there as long as he liked. He had seen testator so drunk that he had brought him home in the bottom of the cart. He was very fond of drink. Witness did not consider a man drunk who could sit up in a cart.

The Judge Ordinary.—I have heard a man say he did not think a man drunk who could lay on the ground without holding. (Laughter.)

Mr. John Terrington, examined by Mr. Serjeant Pigott.—He lived at Wisbech, and was uncle to the deceased. Before he went to America the first time, the deceased went to the Board of Guardians for relief. Witness was at the mother's the same afternoon, when Mr. Bays, a guardian, informed her of the application. The mother said she did not care what became of him or where he went to, they would not give him any money. The father said they must allow him something. On his return from America, he slept the first night at witness's house. He was then the worse for drink. Afterwards he used to come to Wisbech market, the same as other people, and call upon witness. He always talked intelligibly and rationally. He once told witness that his mother said he ought to leave her part of his money.

Cross-examined by Dr. Phillimore.—Witness was on friendly terms with his nephew before his death. He was a drunkard, and his appearance was that of a drunkard. Witness kept a public-house at Wisbech.

Mr. Henry Terrington, farmer, examined by Dr. Tristram.—He was an uncle of the testator, and saw him after his return from America. He talked sensibly.

Mrs. Margaret Bradley, examined by Mr. Serjeant Pigott.—She was a widow living at Emneth. She had known the deceased twenty years. She was accustomed to work at Zachariah Terrington's. She was there on Saturday afternoon, the 21st of January. The testator was out in the grass gound with the little boy. He asked witness how she was, and then told her about his mother attempting to poison him. In the evening Mrs. Terrington sent for her to sit up with the deceased, and give him a drink now and then, as she was poorly and unable to sit up herself. Witness gave him some weak gin-and-water, and weak port wine and water. He took very little. The next morning he was rather worse, and on Monday morning his aunt offered him something to drink, and he turned round on his side, then turned on his back, and went off like a lamb. Mr. Terrington was then gone for the doctor.

Some other letters, written by deceased when in America, were here put in and read.

This was the case for the plaintiff.

The Court adjourned till the next day.

May 6th.

The case for the defendants was proceeded with this morning.

Dr. Phillimore addressed the jury on behalf of the defendants, Mrs. Terrington, the mother, and Mrs. Johnson, the sister of the testator. The grounds of the opposition to the will were in formal language that at the time of the execution of the will, the testator was of unsound mind, and that he executed it under undue influence exercised over him by his uncle Zachariah. In other words, the defendants would contend that the deceased was labouring under imbecility caused by epilepsy and continual and ineradicable drunkenness; that he was also the victim of an insane delusion respecting his mother, who had been excluded from all share in his testamentary bounty. It would also be contended that the party principally benefited in this case, the uncle Zachariah, worked upon this state of the deceased's mind in order to obtain an instrument whereby he was largely benefited. Before coming to the facts of the case, the learned counsel proceeded to state the law upon the subject. He submitted that the mind of the deceased was between a state of absolute idiocy and perfect capacity, and that, according to its degree and amount, the law considered the person suffering under it to be incapable of making his testament. With respect to delusions, the law held, first, that the existence of an insane delusion vitiated a will, whether that delusion was apparent or not at the time of making the will; secondly, that when such a delusion was once proved to have existed, the burden of showing that it had ceased lay upon those who propounded the will. The true definition of a delusion, as given by the law, was this,—a belief of things as realities which existed only in the imagination of the patient.

The Judge Ordinary said the existence of the notion with reference to the mother might not have been a delusion. It must be proved that it was a delusion.

Dr. Phillimore said he was prepared to prove that it was a delusion. At present he should contend that the notion prevalent in the deceased's mind that his mother was seeking to poison him, was to all intents and purposes an insane delusion. The learned counsel then entered into the history of the case, and with reference to the alleged harsh treatment by the mother at an early period of the testator's life, he remarked upon the omission of any evidence in support of that charge, and further submitted that the bestial habits of intoxication which the testator had acquired—which his mother had not taught him, for he had been brought up by his grandfather—rendered it impossible that he should live under his mother's roof. That these habits had not been abandoned upon his return to England was abundantly shown by the evidence which had been given. He was taken to his uncle Zachariah's house in the bottom of a cart; and it would be shown that when drunk he was a dangerous lunatic, and when sober he was perfectly imbecile. In addition to epilepsy, he had had a slight attack of palsy, and he would call evidence to prove that so far from being rational and of sound mind, he was perfectly incoherent in his conversation, and that he was desirous to make a will under the notion that some person would take his property away unless it was secured to him in his lifetime by executors and trustees being appointed to take care of it through the instrumentality of a will. It would also be shown that he had the notion that if he put his money into a bank he could never get it out again; that he could not count his money; and that he did not know the days of the week.

Besides this, the learned counsel referred to the testator's filthy bodily habits as evidence that the brain was affected; and with regard to the conduct of his mother in mixing something in a glass, it would be shown that a medical gentleman had advised her not to deprive him of spirits suddenly, for fear of the consequences, but to gradually reduce the quantity taken by diluting it with water; while at the same time he ordered her to let him have opium occasionally, the testator having been a determined opium-eater. This might be the foundation for the opinion he had formed that his mother was attempting to poison him, for it would be shown that the existence of a poison pill was entirely a delusion. Another fact that would be given in evidence was that the testator made three attempts to make his will, and that the solicitor to whom he applied in each case, being perfectly satisfied that he was incompetent, refused to comply with his request. Having commented upon the case for the defendants at some length, the learned counsel called the following evidence:—

Mrs. Mary Terrington, examined by Mr. Keane.—I am the mother of the testator. I recollect his going to America about eight years ago. He was a very bad boy and we could do nothing with him. He always would have drink ever since he was a child. I recollect his return home in November, 1857. Zachariah brought him to my house, saying he could not do anything with him, and that he could not have him. Zachariah said, "Unless he is well looked after he will be lost;" and that we must either take his clothes away or lock him up, or he would get out in the morning before we were up, and he would be lost. On that occasion, when he came back, my son was drunk. At that time I kept the Jolly Farmers. He continued to live with me while I kept the Jolly Farmers. Upon leaving the Jolly Farmers at the end of February, 1858, we removed into the house adjoining; my son went with us. During the time he was with me, he never thought about anything else but drink. If he was not asleep, he was always drinking, from morning to night, and at night also. He would have drink by the bedside; he never went to bed without. Sometimes he would be a-bed for nearly a week together; sometimes for two or three days. He was very filthy in his habits on these occasions; he used to soil the bed and wet the bed five nights out of seven. He was not clean when out of bed. He never dressed or undressed without assistance; he never washed himself or combed his hair. He used to have vermin, and the girl used to comb his head for him. He used to drink porter when he first came home, then beer, and then brandy. He would get up sometimes four and five times in the night; we could not satisfy him; he either wanted drink, or he wetted the bed, or had one fancy or another. One week I kept account of the quantity he consumed—it amounted to six bottles of brandy, one bottle of rum, and one of gin, besides porter and beer. He would have three pints of brandy and water mixed for him at night. On one occasion I tried to prevent his having any more, and he requested the girl to go upstairs and put his things on. When he came down he said he wanted some brandy-and-water. I told him I wished he would drink something else. He said he would have some brandy, and he reached across and got the poker from my side to knock me over the head. He fell down in reaching for the poker. I got up and ran into another room, and he crawled on his hands and knees and followed me. He drew himself up again by the parlour door, and I and the girl ran out. This occurred one Saturday after we left the Jolly Farmers, some time in April. He was very frequently violent towards me. If we did not let him have the drink he wanted upstairs he would break everything. One morning Zachariah came with a letter from my daughter in America. I asked whether he would have a drop of anything to drink. I said, We have had a bad night with William; he has broken every jug in the house. The testator could not count money; he did not know a sovereign from a half-sovereign. Sometimes he would eat scarcely anything for two or three weeks together; sometimes he would be very ravenous (once or twice in the month), when he would have victuals seven times in the day. I have seen him sleeping in the straw-yard and in the sties out of doors, and sometimes in the chaff-house. On these occasions he was always drunk. In April, 1858, I remember his being away at the Chequers. Zachariah came to me and said they were going to get the girl at the Chequers to marry him; that they were going to take him to Lynn to turn his money over to that girl; and that then they would finish him off as the other was, and then fly about and spend his money. He left me on Monday, the 17th of January. I made him a basin of gruel for his breakfast with some brandy in it. He did not take it; he said he felt he should throw it up. He had some tea and some meat. He said nothing to me, and left me without provocation. On one occasion—the Friday night before he went away—he ground his teeth at me and raised a stick, and said he had a good mind to split my skull. I said, "William, what is this all for?" He said, "You've been after poisoning me." I said, "How can I poison you when I have no poison in the house of any sort."

The Judge Ordinary.—Did he say anything about what you were going to poison him with?

Witness.—No; I called the girl, and said, Mary, he talks of my poisoning him; what can it be about? She said, "Nothing at all, only what he has conceited himself." She got him to bed, and I never heard any more about it. On the Sunday night he wanted to sit up to enjoy himself. He had some beer and tobacco, and he sat up till ten o'clock. I then asked him to go to bed, and he said he would, and he went. That was the last night he was at home. He had been attended by Mr. Wallis and by Mr. Ingle. He took medicine, but he would have beer immediately afterwards. I had opium in the house. I used to get a pennyworth at a time made up into about twelve pills. He used to say, "Mother, can't you give me something to sleep?" I said, "I've got nothing, without it be a little bit of opium, and that can do you neither good nor harm." I had given him a pill now and then. If I had given it as frequently as he asked for it, I should often have been giving it to him. He had not a pill two months before he went away. He had altogether about seven before Michaelmas and two after. I never mixed any opium in what he drank. On Friday, the 21st, the son of Zachariah came for his shirt and stockings; I gave them to him. Some time after I left the Jolly Farmers, I recollect Zachariah and his wife coming to me, and Zachariah said of William, "I am well sure he is not in his right mind." He had convulsive fits very often. During the time he was with me he was not in his right mind. He could remember things many years ago; but he could not remember things that occurred within the last ten minutes. He did not know anything at all about business.

Cross-examined by Mr. Serjeant Pigott.—It was his general habit to drink from a child, when he was seven years old. He would get tipsy, and we could not trust him alone. He would always have drink if he could get it. He was along with his grandfather, and he used to watch for our men to take him to Wisbech. My grandfather put him for a year at a boarding-school. He went to his grandfather at four years of age, and remained with him till he was about seventeen. I had a heavy family of fourteen children. During the time he was at his grandfather's, he used to come to my house occasionally, and would have drink.

By the Judge Ordinary.—I should think he was then about thirteen years of age. He would fly to drink in spite of us, and would take so much as to reel about.

Cross-examination continued.—My husband was accustomed to set a bad example in that respect. He made it the worse for me. My son never took his father before the guardians in consequence of his destitute state. My husband used to give the grandfather something to give him. After he left the grandfather he came home. We could not do anything with him; he wanted to go to sea. He lived with us a year or two before he went to sea. After his return from Quebec the first time, he went about working for his uncle Henry, for Mr. Howlett, and others. He was a bad boy, and robbed us, and we turned him out. He took a young mare off the land, and sold it for 17*l.*, and spent the money, and picked our pockets at night. My husband so provided that he had money to provide him with victuals and clothes, though he did not know where it came from. The money was given to his aunt, Mrs. Howlett, who kept a public-house.

Was not your husband ordered to give him something to maintain him by a guardian?—No, never, sir; my husband always provided for him. I remember Mr. John Terrington and Mr. Bays being at our house on one occasion, but nothing was said about allowing the testator something. I was not on bad terms with him when he went to America. He never wrote to me, but to his grandfather. He used to think his grandfather would leave him a fortune. My son John died in 1857. I was on bad terms with him because he took to drinking and wasted his money. I refused to attend his funeral because he would have taken everything from me and turned me into the Union. He was a good boy to me until he took to drinking. He left his property to his son William, in America. When the testator came home from America I was sent for to go to him at Zachariah's. He was in a fit, and they thought he was dying. He was asleep when I got there. He came to my house a day or two after. He walked over with his uncle Zachariah. He did not stay above ten minutes. I believe he did not sit down, though I had not seen him for six or seven years. He seemed to be wild, and was in a hurry to be off. He came to live with me on the following Tuesday, and it was on that occasion his uncle told me to take his clothes away, or he would be lost. He was drunk when he came. We put him to bed, and put about a quart of porter by his bedside, as he said he would have it. He did not know a Sunday from any other day until the girl told him. He generally asked the girl in the morning what day was this. When it was Saturday we used to tell him it was Friday, as we did not want him to go to Wisbech Market. If he found it out he used to be in a rage. He put his best clothes on to go to market, and on Sundays, when they were got out for him. He was washed every morning and his hair brushed out, and we did not care who saw him. The account I kept of the quantity he drank one week was after he took his money from Mr. Metcalfe—600*l.* or 700*l.* He said he would have a frolic. He received the money in a cheque, and also the title-deeds to the land. He brought them home in his hand. He began to drink liquor in February. Up to that time he principally drank beer and porter. He did pay for the liquor he had after he took his money. I do not recollect how he paid for it. When I wanted a little money I used to ask Johnson to let me have it. The testator, if he had ever so much money, would not pay me himself. He had lent his money to Johnson, who paid interest for it. He kept 100*l.* in his own pocket, which he did not lend to Johnson. He kept his money in his pocket, but it was soon gone. I never saw him pay for anything. He did not pay for his board, nor have I been paid for it from the time he took the money. He settled with me up to the time he took his money. I had lent him about 18*l.* for pocket money, and paid for his clothes. I believe he never knew how much I lent him. I once lent him 5*l.* at a time, and I do not believe he knew whether it was a 5*l.* note or a 10*l.* note. The week he drank the quantity of spirits I have enumerated he was in bed, and I and the girl would take it up to him. He would have two or three pints of brandy-and-water mixed at a time, and put on a chair by his bedside. We could never mix it strong enough; he wanted to have it all brandy. At first I put a quarter of a pint of brandy to a pint of water, but he would have it stronger; and then I put half a pint of brandy to a pint of water.

He would drink till he got drunk?—I believe he would drink till he got sober again. (Laughter). He would drink till he fell asleep. He was always comfortable till he went down the fens in Forth's cart, and then when he came back he appeared quite set against me, and I used to think somebody had been saying something against me. I believe Forth made a deal of mischief. I have said to Forth that I thought he ought not to leave his money out of the family. I thought Forth was "egged" on by the other party to get him away from me. I sometimes take opium by the advice of my medical man. The opium was not got into the house on his account. I have been too good by half to him in letting him have drink. On the Friday, when he spoke to me about the poisoning, was after he had been with Forth down the fen; and then when he came home he was fit to murder me. He was drunk. He used to go out for a walk in the day as far as the public-house. I remember he did go down to Mr. Thomas Bellinson's that week. He used to take a drive in Forth's cart twice a week. He once had a fancy to go hoeing. He went to work about half an hour, and he gave his hoe away. Sometimes he went to market.

Mr. Thomas M. Wilkin, examined by Dr. Phillimore.—I am a solicitor at Lynn, and one of the coroners of the county of Norfolk. I have been in practice twenty years. I know the last witness. I remember in January, 1858, meeting her at Mr. Metcalfe's, Wisbech. The deceased was present, and Mr. Metcalfe, Mr. Johnson, Mrs. Terrington, and myself. A sum of 720*l.* was paid over to the deceased, either in notes or a cheque. I took the title-deeds, and at his request gave them to his mother. When this 720*l.* was paid to the deceased he did not seem to know the value of it. He put the money into his outside pocket. At the Rose and Crown I endeavoured to explain to him the amount of it, and asked what he was going to do with it. He said he did not know. Mrs. Terrington said it would be better to let Mr. Johnson have 600*l.* of it, for which he was to give a promissory note. Mr. Johnson did so, went for a stamp, and I filled up the promissory note, made payable at five per cent. interest. At his request I handed the promissory note to his mother. During this time he was drinking brandy-and-water. He went with Johnson to the bank, and I advised him to put the balance into the bank; and he said, "I can't get it out again if I do." I advised Mr. Johnson to put the balance into the bank on his account, and on their return I asked the deceased what he had, and he said he did not know. I said, "You had better take it out. His mother wanted him to pay some money he owed her for clothes. I told him he had better pay her, and he took the money out of his pocket, and did not know the sovereigns from the half-sovereigns. He then said, "I want a will." I saw he had taken two or three glasses of brandy-and-water, and I said, "You had better come to my office at Lynn, and bring Mr. Johnson with you." He came on the 22nd of February, and he wanted a will. Mr. Johnson was with him part of the time. The deceased said he knew, unless it was done directly, the property would be all gone. I explained to him that it would not, and I asked him what property it was. He said he did not know. I asked to whom he wished to give it. He said he wanted executors to protect the property. I saw he evidently did not know what his property was, and I told him to see me again. He said there was somebody after his property, who had followed him that morning. He came to me again alone in April, and said his uncles were trying to catch him; that they had followed him in a gig to Lynn, and that he was sure the property would go unless something was done. I again asked, "Who is to have your property?" He said, "Mother, Mrs. Desborough, and Mrs. Johnson." I said I would see Mr. Johnson about it, and he went away. He came again in November in a most dreadful state of tremor. I observed the water running from his trousers. I called his attention to it, and he was perfectly unconscious of it. He could not hold a conversation with me for five consecutive minutes upon any subject. On the occasion when I met him at Mr. Metcalfe's, he made a noise, and said he wanted some drink.

Cross-examined by Mr. Serjeant Pigott.—On the occasion when he could not tell the difference between a sovereign and a half-sovereign, he had taken, I should think, two glasses of brandy-and-water, which he drank ravenously. He was never sober when I saw him. On the occasion in question he was not sober when we went to the hotel. Mr. Metcalfe paid the money into William Terrington's hand. There was a release executed by William Terrington. It was not read to him. There was a receipt endorsed on the back. I am not sure how the money was paid, but I remember there was a cheque. He had about 40*l.* when he came back from the bank. On either of the occasions when he came to me he was not in a fit state to make a will. He was under a strong delusion that his uncles were after him. Lynn is about ten miles from where he lived. I had to arrange with a gentleman about some rents due to him, and I remember I could not make the testator sensible about it.

Mr. Joseph Johnson, examined by Dr. Phillimore.—I am the husband of one of the sisters of the deceased. I have been married twelve years. I first saw the deceased after his return from America about the first week in November. I saw him on the road. I stood against the Jolly Farmers. He was coming that way. I considered that he was drunk. He beckoned to me with his hand, and said, "Come, and go with me." I asked where he was going, and he answered he was going coursing. I said, "You have nothing to course with." "Oh," he said, "never mind; if I can find them I can catch them." His mother then came up, and said, "This is Joseph, that married your sister." He came up to the door, and we went into the house. He called for some drink, and I remember there was a man present whom the deceased wanted to fight. By his mother's advice I took him home with me, and as soon as he got into the house he had a fit, which lasted a quarter of an hour. When he recovered from the fit he laid down on the sofa, and fell asleep. My wife came home; and when he awoke he said, "Oh, here's my sister." He sat and talked with us in the evening, and when he went to bed he threw himself on the bed in his clothes, and had another fit. The next morning we went to Wisbech, and on our return I left him at his mother's. On a subsequent occasion I went with him to Mr. Metcalfe's. The testator said he had come to hear about his money. Mr. Metcalfe said the things could not be settled yet. I said there is some of the land uncultivated, and Mr. Metcalfe said he might take possession of that; and the deceased said I might have it. On a third occasion I went with him to Mr. Metcalfe's, and Mr. Metcalfe drew out the lease for seven years. I was also present when Mr. Wilkin settled the affairs of the land in January. Mr. Metcalfe paid the deceased a cheque for 700*l.* or better. He put the cheque into his pocket, and I went with him to the bank; 600*l.* was put to my account, 80*l.* was put to his own account, and he drew a cheque for the remainder. He said he did not like to put money into the bank, because he was afraid he should not be able to get it out again. He did not appear to me to understand the value of money. I farm about 500 acres of land; two-thirds of it is my own, which has been in our possession a century. I hire some land of his mother, and I also hired the deceased's thirty acres, at 2*l.* an acre. He said his uncle was always after him, and he was afraid he would get his money. I saw the corpse of the deceased after he was dead, and I remember Mrs. Zachariah Terrington saying that her boy slept with him for a night or two, but that he did not dare to sleep with him any longer. The deceased slept at my house three times. I paid the deceased money from time to time; sometimes five shillings, sometimes ten, sometimes more. I have paid his mother money on his account. On one occasion I paid her 18*l.* for clothes, shortly after he had taken his money. I know Bellinson; he told me he did not believe that any man who drank like the deceased was fit to do business. I saw Mr. Tubbs on the day of the funeral. He told me, when he attended the deceased at Upwell, the deceased said his mother was going to do for him. In my opinion I did not consider the deceased was in a state to make a will. When I used to give him money, he used a few hours after to say that I had not given him any. That happened several times.

Cross-examined by Mr. Serjeant Pigott.—I did not attend the funeral. I went the night previous. I had heard that there was a will made, and I asked Mrs. Zachariah and the son whether there was a will, and they never spoke. I learned the fact from Mr. Zachariah. I had been to Mr. Metcalfe's to hear about it, my wife having told me she fancied there was a will made. They said they were going to bury him the next day, but they never invited me to attend. When I say he drew a cheque, I mean the bank gave him a deposit receipt for the balance, which he left in the bank to his own account. He also received some 30*l*. or 40*l*. in gold and silver, and he put it into his pocket. I think he put it into his outside pocket. He said his uncles were always after him. I said, "It's only your imagination." He said I did not know so much of them as he did. He said on other occasions that they wanted to get his money.

By a Juror.—I was to pay the rent for the thirty acres quarterly. I have paid rent and have a receipt for the money.

By the Judge.—I took the land at Michaelmas after the deceased came home. I did not pay him quarterly because he said he would rather have the money when he wanted it. I paid him five shillings at a time, and ten shillings, and so on. I have one receipt for the first half year's rent. I have not got it with me, because my solicitor, Mr. Wilkin, told me he thought it would not be required. I mean that he said I need not bring the accounts. I don't think I asked him about the receipt particularly.

The Judge Ordinary.—Another time a solicitor gives you such advice don't obey it; it is much safer.

Mary Fundry, examined by Dr. Phillimore.—I am servant of Mrs. Terrington, the mother of the deceased. I went to live with her the Michaelmas before last. I remember the deceased coming to her house with Mr. Zachariah Terrington. He looked very ill. He went away with Mr. Terrington. He came back on the Tuesday following and was then tipsy. His person was very dirty; he had some things in his head. I always washed him. There was another servant in the house named Charlotte Peck. Sometimes he was violent. I never heard him talk rationally. He used to be bad in bed. I have seen him have fits. He had one the first night he came home. I have seen him have three of a night. Sometimes he used to swear at his mother; sometimes he would say, "You aint half a bad one, don't take notice of me." I remember Zachariah Terrington saying that the deceased was not in his right mind. He said it three times. The deceased never said anything to me about his mother's treatment or about what she had given to him. On Friday night I heard him tell his mother, after he had been out with Mr. Forth, that she was after poisoning him. I heard him say so once before in the public-house. I asked him how he came to think so. He said he did not know. I am sure he did not know the value of money, because when she used to give him money of a morning he would say shortly afterwards, "Mother, give me some money." She would say she had. He replied, "It's a lie." I told him to feel in his pocket, and he would turn his pocket inside out and the money would fall out, when he would say, "Damn it, there is some money; I didn't believe you, you are such liars." On one occasion he asked me to count some money for him, and I counted it for him three times, and he didn't know how much there was. He used to ask me every morning what day it was. I used to dress and undress him. He never said anything to me about his mother putting anything into his drink, nor did I say anything to him about it. I never had such a thought.

Cross-examined by Mr. Serjeant Pigott.—I used to tell him lies sometimes, when there was a fair or anything of that kind, and we did not want him to go for fear he should be lost. He never came home but what he was tipsy. I went to hear the will read, but did not hear it read. He told other people he would put me down in one corner of his will. I never said anything to the effect that I was glad he did not die in our house, or else it might be said I poisoned him. I never said that his mother put something into his drink. I never heard that he said I did. I remember one night she called me in and said, "Mary, come here; William says I've been trying to poison him." I told "missus" not to take any notice of it, for he would say anything. I don't know what my "missus" said; I know she was finely hurt about it. I say he never talked rationally, because he would run on such a many things. He would say sometimes, "I want to go to such a place." I used to get out his best clothes for him; he never told me to get them out. He would say, "I am going with Mr. Johnson." I very often used to take brandy upstairs for him. I used to take up half a pint of brandy and put it behind his pillow; and then I used to get up in the night and give it to him. He would make such a noise, calling out, "I want something to drink," and he could not find it. He would break the mugs, and everything in the room.

By the Judge.—I used to tell him not to drink so much or he would kill himself. I used to mix his drink for him at night. I never could make it strong enough for him. Once he hurled some at me because it was not strong enough. He complained several times that his mother did not mix it strong enough, and he would make me take it down again. I never told him I had thrown it away and mixed him some more. I used to put some more brandy to it, and then he would say, "It's damned little you put to it." I used to say, "It is not my fault, I didn't mix it; it's no use grumbling with me, your mother mixed it for you." When he was at the public-houses I used to say he would poison himself drinking so much brandy. He would say he didn't care.

James Morris Miller, examined by Mr. Keane.—He was the landlord of the Jolly Farmers, and succeeded Mrs. Terrington. He saw the deceased for the first time last January twelvemonth. After he took the house he saw the deceased every day that he could get out. He used to be generally drunk; that was his general habit. He had seen him lying down with the pigs in his yard; he was in such a dirty state that he did not look like a human being—he was wet and dirty. At the best of times he did not appear to be a rational man. Witness did not believe he could attend to business. In paying for his drink, if he gave half a sovereign or a sixpence, he never knew when he got the right change. On one occasion, a day or two before he left home, he said he thought he should be poisoned. Witness told him he was a foolish chap; he got drunk; and did not know what he ailed. Deceased offered to sell him land once, and when asked at what price, he said, "Damn it, what do I know about it;" and when asked whether it was copyhold or leasehold, he said with an oath, "What should I know about it."

Cross-examined by Mr. Serjeant Pigott.—The deceased used to drink at his house, too much to his sorrow. He could not keep him from having drink; if he refused to serve it, the deceased would kick up a row. Witness always gave him his right change. Mrs. Terrington was the owner of the Jolly Farmers.

Henry Cott, examined by Mr. Keane.—He was a farmer at Marshland Smeeth. He recollected the deceased's return from America. He was always drunk, and did not appear to be fit for any kind of business.

Mrs. Mary Ann Clarke, examined by Mr. Keane.—Her husband was the landlord of the Smeeth Station Inn. The deceased called sometimes every day. She had wondered how he would appear when sober; she never saw him sober; was very dirty in his habits, and did not appear to be conscious of it. His memory was defective. He used to say that he was going to alter his will, and he would leave her child a fortune. Sometimes he said he would divide it equally between his mother and two sisters. Sometimes he promised to leave land to persons in her house. When she had taken a shilling from him in payment of beer, and had fetched the change from another room, he appeared to have forgotten all about it, and asked what it was for. He did not seem to know the difference between halfpence and pence, or between a sixpence and a shilling.

Cross-examined by Mr. Serjeant Pigott.—On these occasions he was tipsy, or if not tipsy he was in a very strange state of mind. He conducted himself in her house in a strange way, and would sometimes express in the most impious terms a wish that his legs might drop off.

Mr. William Pike Bays, examined by Dr. Phillimore.—I am a conveyancer at Wisbech. I attended the funeral of the deceased. I saw Mr. Ollard there and had some conversation with him. I expressed my surprise that he had made the will; and he said he did not like it, and he thought he should not act. I knew the deceased several years ago, before he went to America. I have seen him only once since his return.

Cross-examined by Mr. Serjeant Pigott.—I attended to have the will read at the request of Mrs. Terrington and Mrs. Johnson. I told Mr. Ollard the will would be disputed. He said he was pressed to be a trustee, and I thought I would call in Mr. Tubbs as to the competency of the deceased to make a will. Mr. Ollard said he did not like it, and he thought he should not act. That was before I said the will would be disputed. I made a minute of the conversation, and I find in that minute, "Mr. Ollard said he intended to renounce; he didn't much like the business."

Mr. John Gathergood, examined by Dr. Phillimore.—I am a general practitioner at Terrington St. John's, near Wisbech. I have seen the deceased repeatedly up to within a very short period before his death. I had opportunities of forming an opinion as to his mental condition. I considered him a person of weak mind, and not competent to do an act that required thought, memory, and understanding.

By the Judge Ordinary.—By that I mean I never saw him in a condition to transact business properly.

Examination continued.—He showed the weakness of his mind by the incoherent manner in which he spoke, and the unreasonableness of the matters which he brought under my notice. He has called at my residence to consult me professionally, and in attempting to detail the history of his case he has made remarks of a very irrelevant character. He especially on one occasion seemed to have a sort of delusion that he could see things that did not exist. On one occasion he said, "Look there, Gathergood, there goes a man with a ball of fire on his head as big as a bass drum." He also said his mother was attempting to poison him, and that the servant girl had repeatedly thrown the poison away. He said he felt very uncomfortable in his mind, because he was afraid that some of his relatives would deprive him of his property. He asked me to go with him and have his will made. I refused, as I did not think it was a matter that concerned me. He has spoken to me about his death. He said if he died at his mother's residence, he hoped I would cause a coroner's inquest to be held over him, for he was sure she would poison him. With regard to the state of his health, on the first occasion of his consulting me he was suffering from syphilis. He was subject to incontinence of urine, and maladies of a similar nature. He was subject to epileptic fits. His appearance was that of a person totally emaciated from irregular habits. I considered he was partially suffering from palsy. He spoke to me about his property. He said he should leave some to his sister in America, and the remainder to Mrs. Johnson. He expressed a desire to leave my son 100*l*. I believe he was a person of that character that any person could control him at the time.

Cross-examined by Mr. Serjeant Pigott.—I married Mr. Johnson's sister. Habits of intoxication may be as common in our neighbourhood as in other parts. I have been given to them occasionally.

By the Judge.—I have heard him say repeatedly that his mother was attempting to poison him. Last June was the first time. It was about last Michaelmas when he asked me to have an inquest held should he die at his mother's house.

Dr. Forbes Winslow, examined by Mr. Keane.—Intemperance is a common cause of insanity.

The Judge Ordinary.—Some people go mad from drink; that's the whole of it.

Examination continued.—A craving for drink is not always a proof of a diseased state of the mind. Epilepsy, in conjunction with intemperance and delusions, would be a symptom of a diseased state of the mind.

The Judge Ordinary.—Dr. Forbes Winslow is assuming the existence of delusions.

Examination continued. —Incontinence of urine and other matters indicate a want of control over the parts, and would lead to the suspicion that the mind was affected; it might exist, however, without disease of the brain.

The Judge Ordinary.—Assuming all those symptoms existed, and the person in whom they were observed should go to an attorney and hold a continuous conversation with him, and explain to him his views about his property distinctly, and express in a distinct manner the disposition which he meant to make of it, and to reason upon it, and he is remonstrated about not leaving it to his mother and sister, and he says he is resolved he would not, whatever might be the consequences, would you say those symptoms would under such circumstances indicate insanity?—Certainly not.

The Judge Ordinary.—You see, gentlemen, it is merely a speculative opinion.

This was the case for the defendants.

Dr. Phillimore summed up the evidence.

Mr. Serjeant Pigott replied upon the whole case. He would not question for a moment that the deceased was an habitual drunkard. The question they were trying was as to the validity of this will. It had been attacked on two grounds. With respect to the exercise of undue influence by his uncle, there was not a tittle of evidence. The man came from America, and went to his uncle's house, and they instantly sent for his mother; and it was by their advice that he called upon her the next day. So little did Zachariah Terrington desire to interfere that he remained outside while the interview lasted. The son remained in the house only ten minutes, and, considering that she had not seen him for six years, that of itself was a proof of the state of feeling that existed between him and his mother. He afterwards went and lived with his mother for twelve months, and during the whole of that period there was no proof that the uncle ever attempted to exercise any influence whatever over him. The mother said they were on good terms with uncle Zac. That part of the case, then, might be dismissed. Then they came to the more important ground of opposition—that the testator at the time he made the will was in an unsound state of mind. It must be for the jury to say whether on that 19th of January, when he went to Mr. Ollard's office and gave the instructions he did as to the distribution of his property, and showed a full knowledge of the nature of his property, and of the persons to whom he wished to leave it, and conducted himself in the sensible and rational manner which had been spoken to both by Mr. Ollard and Mr. Tubbs, whether upon that occasion he was not in a state of mind competent to make a will? The learned serjeant then reviewed the evidence adduced on the part of the defendants, and submitted that the conduct attributed to the testator by the various witnesses, and his acts of folly and weakness, might be attributable to his drunken habits, but that they were no proof of insanity.

The Judge Ordinary summed up at great length. He said there were two points for consideration. First, whether the testator had sufficient understanding to dispose of his property at the time he made his will, and secondly, whether he was under any undue influence at the time. He would dispose of the latter question first. Undue influence did not mean persuasion and cajoling, by pretended kindness, or anything of that sort, but it meant the exercise of such control as to induce the party to make a will otherwise than he wished it, because he was controlled and urged in that manner so that it was not his will but the will of somebody else. Now, in this case there certainly was no evidence of any undue influence of that sort. The man went with his uncle into the office of the attorney, and there was no proof of any attempt to control on his part. The case resolved itself into the question whether he had capacity enough to make a will at that time. This again divided itself into two questions. A man was said to have sufficient capacity to dispose of his property by will if he had mind enough to understand his position, the nature and amount of his property, to remember his different relations, and to form a judgment as to those who should be selected as objects of his bounty. If he had sufficient understanding for that, he had enough to enable him to make a will. But the understanding, although limited in its range, must be a sound understanding. A person who was mad on half a dozen points might have sufficient capacity to know his property and to prove an intention as to who should have it after him. His will would not stand good because the mind was unsound. So if in this case the man had any insane delusion upon his mind, and acted under the influence of an insane delusion, that mind was unsound, and they could not maintain a will that was made by a person under those circumstances. If he acted under a mistaken belief that certain things had been done or said of him or towards him, although it might be that such things never were said and never were done, if he believed that seriously, and acted upon that mistake, that would not invalidate his will. But if it was an insane imagination, not formed upon any ground whatever, but a mere conceit of his own, not founded in reason, but proceeding from insanity, then undoubtedly the existence of such a delusion would invalidate the will. The case most frequently cited was the case of *Greenwood*, a barrister who had been undoubtedly insane. He recovered so far as to follow his profession for many years, but the impression that his brother intended to poison him was never effaced. That insane delusion remained to the last; there was no doubt that the origin of it emanated from insanity; that was an insane delusion that pursued him through life, and his will was not established. In the present case there was a statement that the man had got it into his head that his mother intended to poison him. He assigned as a reason for it that the girl told him so. If the girl had said so, or if any other person had told him that the girl had said so to somebody else, and he having heard that, although it was an absurd thing to believe, yet did believe it, there would be no proof of insanity there. It might show a weak mind; it might show a person liable to be imposed upon; and if they could trace that imposition to any person who imposed it upon him for the purpose of procuring a will, and the will was made under the influence of the imposition, that would invalidate it; that would indeed be undue influence, and would be recognised, as in a case in the House of Lords, as sufficient ground for setting aside the will. With these preliminary remarks, the learned Judge proceeded to comment upon the evidence in detail as given on the respective sides.

A Juror.—If the deceased made a will under the delusion that his mother intended to poison him, would that invalidate the will?

The Judge Ordinary.—If it was an insane delusion it would; if it was a misapprehension, a mistake, a suspicion, it would not.

The jury retired, and after remaining out of court about ten minutes, returned with a verdict in favour of the plaintiff upon all the issues.

The question of costs was left for future consideration.

